# 889 Periorbital Burn Injuries: Patterns in Prevalence, Consultation, and Management

**DOI:** 10.1093/jbcr/iraf019.420

**Published:** 2025-04-01

**Authors:** Artur Manasyan, Michael Kim, Nicolas Malkoff, Brigette Cannata, Sarah Wang, Maxwell Johnson, Haig Yenikomshian, Justin Gillenwater

**Affiliations:** Keck School of Medicine of University of Southern California; Keck School of Medicine of University of Southern California; Keck School of Medicine of University of Southern California; Keck School of Medicine of University of Southern California; Keck School of Medicine of University of Southern California; Keck School of Medicine of University of Southern California; Keck School of Medicine of University of Southern California; Los Angeles General Medical Center

## Abstract

**Introduction:**

Periorbital burns pose significant risks for complications such as contracture, infection, and ocular damage, potentially threatening vision. In this study, we aim to fill the gaps in the literature by examining the prevalence, characteristics of presentation, and consultation patterns of periorbital burns.

**Methods:**

A retrospective review was conducted for all patients admitted to a large urban burn center between January 1, 2023 and December 31, 2023. Admission records were screened to identify burns involving the periorbital region. Data on patient demographics, hospital course, and outcomes were collected. Descriptive statistical analysis was utilized to report findings.

**Results:**

Among 823 admitted patients, 170 (20.7%) patients had periorbital involvement, 73.5% of whom were male. The average age was 39 ± 20.2 years. Eyelid burns were present in 74.0% (n=125) of patients, most of which were superficial partial-thickness burns (65.8%, n=82) and mixed partial-thickness burns (20.7%, n=26). Ophthalmology consultations were obtained for 71.8% (n=122) of patients, with an average time of 16.7±26.5 hours from admission until consultation. There were no cases of ealy ectropion, while late ectropion occurred in 2.1% of cases. Early corneal injuries were reported in 33.3% of cases, with 4.8% experiencing late corneal injury or scarring. Vision loss on admission was present in 11.3% of patients, with 7.0% experiencing long-term vision loss after discharge. Increased intraocular pressure (IOP > 21 mmHg) was observed in 21.3% of patients, and orbital compartment syndrome occurred in 2.0%. Surgical interventions included eyelid surgery in 8% of cases, debridement in 7%, split-thickness skin grafting in 4%, and full-thickness skin grafting in 2%. Medical management commonly included topical lubricants (63.8%) and topical antibiotics (49%).

**Conclusions:**

Periorbital burns are prevalent yet under-researched, often leading to chronic complications such as vision loss and corneal scarring. Although a majority of patients received ophthalmology consultations, it is essential that nearly all individuals with periorbital involvement have the ophthalmology team integrated into their care early on.

**Applicability of Research to Practice:**

Burn surgeons should prioritize early collaboration with ophthalmology colleagues for patients with periorbital burns to effectively mitigate chronic complications like vision loss and corneal scarring.

**Funding for the Study:**

N/A

